# Psoas Major muscle area as a prognostic marker in peripheral arterial disease: a systematic review and meta-analysis

**DOI:** 10.3389/fsurg.2026.1879694

**Published:** 2026-07-15

**Authors:** João Fernandes-Carvalho, João Braga-Simões, Daniela Santos-Silva, Vítor Sá-Martins, José Paulo Andrade, Hugo Ribeiro, João Rocha-Neves

**Affiliations:** 1 Faculty of Medicine of the University of Porto, Porto, Portugal; 2 Unidade de Saúde Familiar - UarcoS, Unidade Local de Saúde do Alto Minho EPE, Viana do Castelo, Portugal; 3 Life and Health Sciences Research Institute (ICVS), School of Medicine, University of Minho, Braga, Portugal; 4 ICVS/3B's-PT Government Associate Laboratory, Braga/Guimarães, Portugal; 5 Department of Palliative Care and Pain Medicine, Faculty of Medicine, University of Coimbra, Coimbra, Portugal; 6 NOVA Medical School | Faculdade de Ciências Médicas da Universidade Nova de Lisboa, Lisboa, Portugal; 7 CHRC - Comprehensive Health Research Centre, NOVA Medical School | Faculdade de Ciências Médicas, Universidade Nova de Lisboa, Lisboa, Portugal; 8 Department of Biomedicine - Unit of Anatomy, Faculty of Medicine of the University of Porto, Porto, Portugal; 9 RISE-Health, Departamento de Biomedicina, Faculdade de Medicina, Universidade do Porto, Porto, Portugal; 10 Faculty of Medicine of the University of Coimbra, Coimbra, Portugal; 11 Community Palliative Care Team Gaia - Local Health Unit Gaia and Espinho, Vila Nova de Gaia, Portugal; 12 Coimbra Institute for Clinical and Biomedical Research, Coimbra, Portugal; 13 Department of Vascular Surgery, Unidade Local de Saúde - Alto Ave, Guimarães, Portugal

**Keywords:** frailty, mortality, peripheral vascular diseases, sarcopenia, vascular surgical procedures

## Abstract

**Introduction:**

Although frailty and reduced muscle mass are known to worsen surgical outcomes, the prognostic value of psoas major morphometry in peripheral arterial disease (PAD) remains incompletely defined. Sarcopenia, frequently quantified through cross-sectional psoas major muscle area on preoperative imaging, has emerged as a potential prognostic marker in patients undergoing lower-limb revascularization for PAD. This systematic review and meta-analysis aimed to evaluate whether reduced psoas major muscle area is associated with short- and long-term mortality following revascularization.

**Methods:**

A systematic search of MEDLINE, Scopus, and Web of Science identified observational studies assessing psoas muscle morphometrics in adults undergoing surgical or endovascular revascularization for PAD. Seven retrospective cohort studies (*n* = 2,290 patients) met the inclusion criteria. Study quality was assessed using the NHLBI tool. Random-effects meta-analyses were performed for 1-month and 1-year mortality. Heterogeneity was evaluated using I², and prediction intervals were calculated. Due to the small number of studies, meta-regression was not performed.

**Results:**

Three studies contributed to the 1-month mortality analysis. The pooled estimate suggested a possible increase in early mortality among sarcopenic patients with a smaller psoas major area, but the association was not statistically significant (RR = 2.45; 95% CI 0.58–10.36; *I*^2^ = 38.3%; *p* = 0.22). Five studies reported 1-year mortality, demonstrating a significant association between reduced psoas major muscle area and increased long-term mortality (RR = 2.37; 95% CI 1.51–3.73; *I*^2^ = 43.4%; *p* < 0.001). Although point estimates remained directionally consistent across heterogeneous imaging methodologies and sarcopenia definitions, confidence intervals were wide, and the prediction interval for 1-year mortality crossed the null value, indicating uncertainty regarding the magnitude and consistency of the association across future settings. Insufficient data prevented quantitative synthesis of limb outcomes.

**Conclusion:**

A reduced psoas major muscle area was associated with increased 1-year mortality in patients undergoing lower-limb revascularization for PAD, supporting a potential role for psoas morphometry as a prognostic marker of long-term vulnerability. Although short-term mortality findings were inconclusive due to limited power, psoas morphometry may have value in preoperative risk stratification. Standardized definitions and prospective studies are needed before routine clinical integration can be recommended and to clarify whether targeted nutritional and rehabilitation strategies may improve outcomes.

**Systematic Review Registration:**

identifier CRD420251118644, https://www.crd.york.ac.uk/PROSPERO/view/CRD420251118644

## Introduction

Peripheral arterial disease (PAD) encompasses atherosclerotic disease of the noncoronary arterial beds, particularly those of the lower extremities, as well as the renal, mesenteric, and abdominal aortic circulation (1). Patients with PAD constitute a particularly vulnerable population, frequently presenting with advanced age and a high burden of cardiovascular comorbidities (2, 3). Although surgical and endovascular revascularization techniques have evolved substantially, clinical outcomes remain suboptimal in a significant proportion of patients, with persistent risks of perioperative complications, restenosis, limb loss, and mortality (4–6). These unfavorable outcomes suggest that revascularization alone may not fully address the complex pathophysiology of PAD, underscoring the importance of patient-related factors that may influence prognosis following invasive treatment.

The cross-sectional area of the psoas major muscle, typically measured on abdominal computed tomography at the level of the third or fourth lumbar vertebra, has emerged as a reliable imaging biomarker for sarcopenia (7). Because psoas major is a core muscle with relatively consistent anatomical boundaries, its area provides a practical proxy for overall skeletal muscle mass, particularly in settings where full-body composition assessment is not feasible (8). Furthermore, conventional sarcopenia assessment often relies on measures of muscle strength and physical performance, which may be difficult to obtain in patients with advanced peripheral arterial disease due to impaired mobility and ischemic symptoms (9, 10). Reductions in psoas major muscle area have been strongly correlated with frailty, decreased functional capacity, and poorer clinical outcomes across a range of medical and surgical populations (11–14). As such, quantifying the psoas major area offers a simple, reproducible, and clinically meaningful approach to identifying sarcopenia and stratifying patient risk in both research and routine practice.

This study aimed to systematically review whether the psoas major muscle area is a prognostic marker of clinical outcomes and mortality in patients with peripheral arterial disease undergoing revascularization. It also aimed to synthesize existing evidence to evaluate its potential role in preprocedural risk stratification.

## Materials and methods

This systematic review was conducted in accordance with the Preferred Reporting Items for Systematic Reviews and Meta-Analyses (PRISMA) Statement and appraised using the Assessing the Methodological Quality of Systematic Reviews (AMSTAR-2) critical appraisal tool (15, 16).

The research question was defined according to the PICOS framework: Population—adults with peripheral arterial disease undergoing lower-limb revascularization; Exposure—reduced psoas muscle area as a surrogate of sarcopenia; Comparator—patients without reduced psoas muscle area; Outcomes—short- and long-term mortality, limb-related events, and postoperative complications; Study designs—observational cohort studies.

Institutional review board approval was not obtained due to the nature of this study. The review protocol was registered in PROSPERO (reference: CRD420251118644). Although the original PROSPERO protocol focused on open aortic revascularization for aortoiliac occlusive disease, the eligibility criteria were subsequently broadened to include all lower-limb revascularization procedures for PAD. This modification was implemented because the available literature was limited within the originally defined population and because the broader population was considered more appropriate for evaluating the overall prognostic value of psoas morphometry in PAD.

### Selection criteria

Inclusion criteria comprised original studies of adult patients (≥18 years) with peripheral arterial disease undergoing invasive treatment that evaluated psoas muscle area as a surrogate marker of sarcopenia. Systematic reviews and case series with fewer than 50 participants were excluded. No exclusion criteria based on publication language were applied; however, only studies published from January 1, 2000, onwards were included.

### Search strategy

A systematic search was conducted across three databases—MEDLINE, Scopus, and Web of Science—in August 2025. The search strategy was based on combinations of free-text keywords related to sarcopenia, psoas muscle morphometry, and peripheral arterial disease, using the Boolean operators AND and OR. The complete search strategy is provided in Supplementary Table S1.

Additionally, the references of the included primary studies were screened for any additional articles of potential interest.

### Study selection and data extraction

After duplicate removal, two authors (JFC and JRN) independently participated in study selection; any disagreements were resolved by a third author (HR). First, studies were selected based on title and abstract; the remaining studies were eligible for full-text assessment. Efforts were made to contact the authors to obtain the full texts that were not publicly available. The selected studies were screened to avoid overlapping patient populations.

Data were independently extracted by two authors (JFC and JRN) using a purpose-built form, including year of publication, country and recruitment center, study design, recruitment period, number of participants undergoing PAD revascularization, participant demographics, cardiovascular comorbidities, definitions and measurement methods of sarcopenia, mortality outcomes, limb-related events, and postoperative complications.

### Assessment of study quality

Concerning qualitative assessment, the National Heart, Lung, and Blood Institute (NHLBI) Study Quality Assessment Tool for observational cohort and cross-sectional studies (2021) was used (at https://www.nhlbi.nih.gov/health-topics/study-quality-assessment-tools—Accessed 11/2025). This assessment was independently performed by two authors (JFC and JRN). When disagreements arose, decisions were made by mutual consensus following a third-party review (HR). The quality of evidence for the included articles was evaluated using the Grading of Recommendations, Assessment, Development, and Evaluation (GRADE) approach. Articles were classified into four quality levels (high, moderate, low, and very low) (17).

### Quantitative synthesis

A random-effects meta-analysis using the Mantel-Haenszel method was performed to evaluate the association between lower psoas muscle area and all-cause mortality at 1 month and 1 year, generating pooled risk ratios (RRs) with 95% confidence intervals. The primary pooled outcome was 1-year all-cause mortality.

When studies reported psoas muscle area in tertiles or quartiles, the lowest category was compared with the highest category to provide a consistent extreme-group contrast across heterogeneous studies. Intermediate categories were excluded from the primary analysis because outcome data were not uniformly reported or directly comparable. Sensitivity analyses, including leave-one-out analyses, were performed where data permitted.

Heterogeneity was assessed using Cochran's *Q-*test and the *I*^2^ statistic; *p*-values <0.10 and *I*^2^ ≥ 50% were considered indicative of substantial heterogeneity. Prediction intervals were calculated where appropriate to assess the expected range of effects in future settings. Meta-regression was not performed due to the low number of included studies (*n* = 7). Potential publication bias was explored visually using funnel plots.

All statistical analyses were performed using metaanalysisonline.com (accessed November 1, 2025).

## Results

### Search results

**Figure 1:**
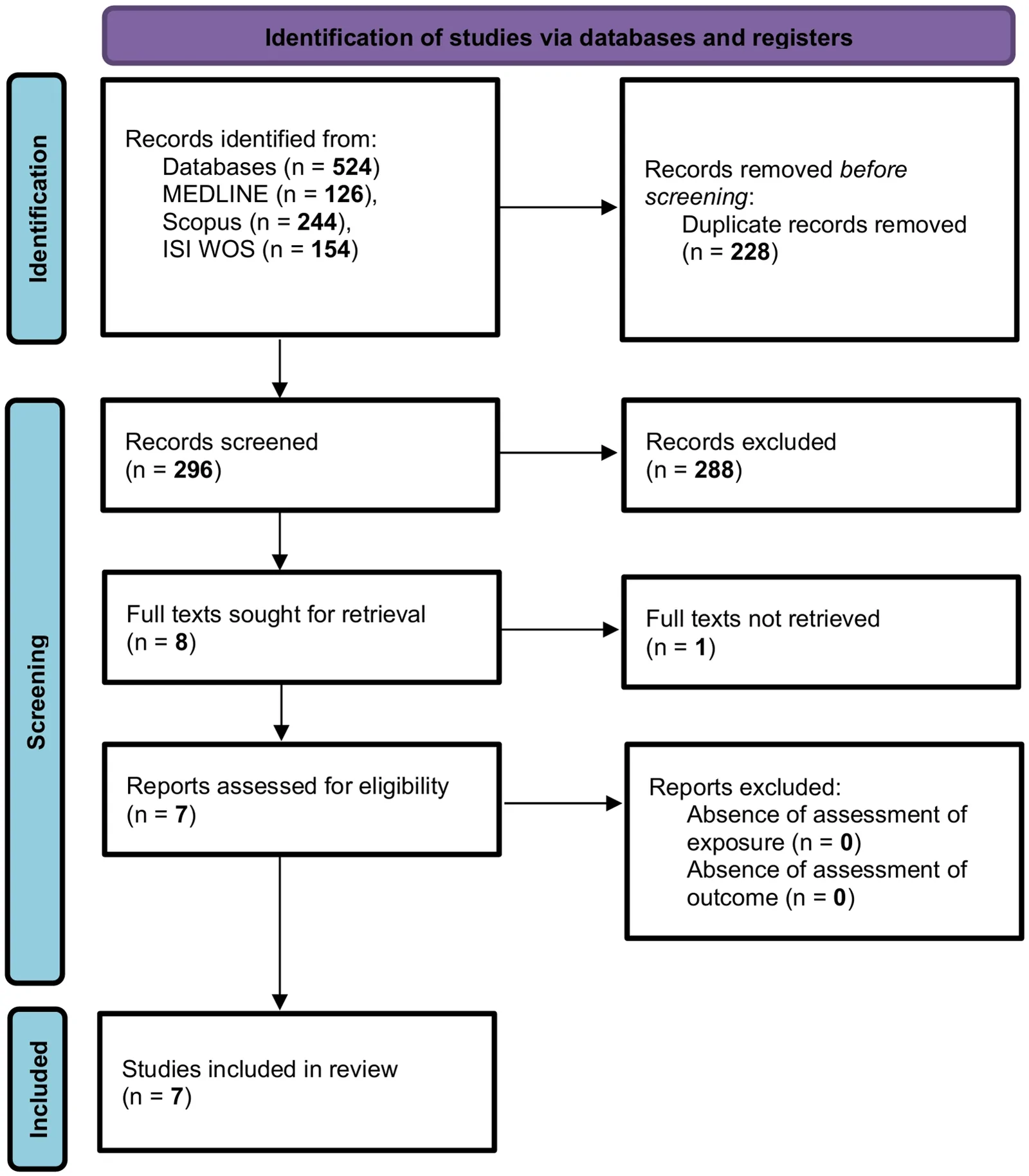
Preferred reporting items for systematic reviews and meta-analyses (PRISMA) flow diagram of the search strategy for articles studying the psoas major area as a predictor of outcomes in lower-limb revascularization.

The literature search identified 524 records, of which 228 duplicates were removed, leaving 296 unique records for screening. Following title and abstract screening, eight articles underwent full-text review. One article could not be retrieved despite attempts to contact the authors, and seven studies met the inclusion criteria and were included in the final synthesis (Figure 1). No studies were excluded due to inadequate exposure or outcome assessment.

### Description of studies

All included studies were retrospective observational cohorts. No randomized controlled trials (RCTs) were identified among the included studies. The included publications were conducted in six different countries across three continents: one from North America (United States) (18), three from Europe (Finland, Portugal, United Kingdom) (19–21), and three from Asia (Japan and Turkey) (22–24).

Across the seven included studies, 2,290 patients were evaluated, predominantly older adults (mean age 60–73 years) with a marked male predominance. Cardiovascular risk factors were highly prevalent: hypertension, dyslipidemia, diabetes and smoking history were consistently prevalent across cohorts, reflecting the typical atherosclerotic profile of this population. Comorbidities such as coronary artery disease, chronic lung disease, and chronic kidney disease were variably reported but appeared common across cohorts. Overall, the demographic characteristics were broadly comparable between studies and representative of patients undergoing lower-limb revascularization and psoas-based sarcopenia assessment (Table 1).

**Table 1:** Demographics of selected studies.

**Author**	**Sample Size**	**Age (years)**	**Male (n)**	**Hypertension(n)**	**Dyslipidaemia (n)**	**Diabetes (n)**	**Smoking History (n)**	**Coronary Artery Disease (n)**	** Heart Failure (n)**	**Chronic Obstructive Pulmonary Disease (n)**	**Renal dysfunction/CKD (n)**	**Body Mass Index (mean or median)**
Chikata et al. (22)	591	71.3 ± 8.9	377	394	354	303	Current 129	NA	NA	NA	273	22.9
Söderlund et al. (20)	899	70 (range 38–93; IQR 13)	569	718	582	330	Former 381 + Current 339	301	NA	NA	NA	26.6
Selçuk et al. (24)	217	61.5 ± 10.9	201	121	56	100	209	103	NA	28	8	Not sarcopenic—26.93, sarcopenic—23.88
Pereira-Neves et al. (19)	57	60 ± 8.2	55	37	37	19	55	14	4	5	Creatinine (>1.5 mg/dL)—7	NA
Taniguchi et al. (23)	75	72.9 ± 10.3	53	60	16	54	(Former + Current) 60	23	NA	NA	NA	Not sarcopenic—23.5, sarcopenic—21.4
Juszczak et al. (21)	263	68.9 (IQR 61.5–75.6)	190	152	NA	59	Former 82 + Current 114	NA	9	69	262	Male—26.4, female—26.1
Nyers et al. (18)	188	66 (SD not reported)	156	160	154	94	Current 75	80	20	28	30	27.2

IQR, interquartile range; NA, unavailable data; SD, standard deviation.

Clinical classification systems and ischemic severity were heterogeneously reported across studies. Rutherford classes were the most frequently used, with several cohorts including substantial proportions of patients presenting with advanced stages (classes 4–6). Two studies also provided Fontaine distribution or Wound, Ischemia, and foot Infection (WIfI) ischemia grades, confirming a predominance of symptomatic and limb-threatening disease. Baseline ABI values were reported in only one study, indicating severe ischemia prior to treatment and marked improvement post-revascularization. Functional status and the American Society of Anesthesiologists (ASA) physical status classification system were sparsely documented. Exclusion criteria varied substantially—most commonly the absence of adequate preoperative imaging—reflecting methodological heterogeneity across the included cohorts (Table 2).

**Table 2:** Clinical classification systems and patient exclusion criteria.

Author	Fontaine class I/II/III/IV (n)	WIfI ischemic grade (n)	Rutherford class (n)	CLTI (n)	Pre-op ABI	Post-op ABI	ASA score	Functional status	Exclusion criteria
Chikata et al. (22)	NA	NA	Classes 1–2–3: 323	NA	NA	NA	NA	NA	NA
			Class 4: 23						
			Classes 5–6: 128						
Söderlund et al. (20)	2/545/224/128	0:340; 1:213; 2:136; 3:164; Missing:48	NA	NA	NA	NA	NA	NA	Inadequate MRI quality or timing
Selçuk et al. (24)	NA	NA	Class 4: 163	NA	NA	NA	NA	NA	Malignancy, inadequate CT or missing data
			Class 5: 41						
			Class 6: 13						
Pereira-Neves et al. (19)	NA	NA	Class 3: 17	35	0.3	0.77	2.6 ± 0.59	Dependent: 0; Partial: 4; Independent: 53	Non-atherosclerotic etiology
			Class 4: 27						
			Class 5: 9						
			Class 6: 3						
Taniguchi et al. (23)	NA	Am	Class 5: 67	75	NA	NA	NA	NA	Inadequate or unavailable perioperative CT imaging, revision cases excluded
			Class 6: 8						
Juszczak et al. (21)	7/82/88/84	NA	NA	172	NA	NA	ASA III predominant (64.2%)	NA	Unavailable perioperative CT imaging
Nyers et al. (18)	NA	NA	Class 3: 81	NA	NA	NA	NA	NA	NA
			Class 4: 52						
			Class 5: 55						

ABI, ankle-brachial index; ASA, American Society of Anesthesiologists; CLTI, chronic limb-threatening ischaemia; CT, computed tomography; MRI, magnetic resonance imaging; NA, unavailable data; WIfI, Wound, Ischemia, and foot Infection.

Assessment of sarcopenia was heterogeneous across studies, with considerable variability in imaging modality, anatomical level, and cutoff definitions. Most cohorts relied on computed tomography (CT) measurements of psoas area at L3 or L4, whereas one study used MRI. Sarcopenia was generally defined using the lowest tertile or quartile of the psoas index, although two studies applied validated population-specific cutoffs. Measurement techniques ranged from quantification of total psoas area (TPA) to indexed metrics such as psoas muscle index (PMI), psoas-to-lumbar vertebral index (PLVI), and L3 psoas index (LPI). The number of sarcopenic patients ranged widely (from 15 to 225 per study), reflecting differences in methodology and population characteristics. Despite these inconsistencies, all studies operationalized sarcopenia through objective morphometric analysis of psoas musculature (Table 3).

**Table 3:** Definitions and measurement methodologies of sarcopenia.

Author	Interventions (n)	Type of Intervention	PAD distribution	Psoas area methodology	Definition of sarcopenia	Sarcopenic (n)	Imaging modality	Anatomical measurement level	Urgency of procedure
Chikata et al. (22)	591	Endovascular 591	Aortoiliac and femoropopliteal	PMI	Lowest tertile of PMI (PMI < 3.009)	162	CT	L4	NA
Söderlund et al. (20)	899	Open surgery 350, Endovascular 428, Hybrid 121	Aortoiliac and femoropopliteal	PMA	Lowest quartile of PMA	225	MRI	L4	Elective 558, Urgent 312, Emergency 29
Selçuk et al. (24)	217	Open surgery (Aortofemoral bypass 57, Aortofemoral and femoropopliteal bypass 33, Femoropopliteal bypass 120, Axillofemoral bypass 4, Femoro-femoral bypass 3)	Aortoiliac and femoropopliteal	PMI	PMI < 5.5 cm²/m² (men); PMI < 4.0 cm²/m² (women)	82	CT	L3	NA
Pereira-Neves et al. (19)	57	Open surgery 32, endovascular 25	Aortoiliac	TPA	TPA < 2,175 mm²	15	CT	L4	NA
Taniguchi et al. (23)	75	Open surgery 75	Popliteal and distal arteries	LPI	Lower two-thirds of sex-specific LPI distribution	50	CT	L3	NA
Juszczak et al. (21)	263	Open surgery (Aortofemoral bypass 20, Axillofemoral bypass 13, Femorofemoral crossover 18, Femoral endarterectomy 57, Femoropopliteal bypass 69, Femorodistal bypass 33, Iliofemoral bypass 41, Exclusion bypass 12)	Aortoiliac and femoropopliteal	TPA	Lowest quartile of TPA	63	CT	L4	Elective 170, Emergency 93
Nyers et al. (18)	188	Open surgery 98, endovascular 90	Lower-extremity arterial disease	PLVI	Low PLVI	94	CT	L4	NA

CT, computed tomography; LPI, L3 psoas index; MRI, magnetic resonance imaging; NA, unavailable data; PLVI, psoas-to-lumbar vertebral index; PMI, psoas muscle index; TPA, total cross-sectional area of the psoas muscles,.

Reporting of early postoperative outcomes was limited and heterogeneous across studies. Only a minority of cohorts provided stratified data comparing sarcopenic and non-sarcopenic patients. Among studies that did, sarcopenic patients generally exhibited higher rates of graft thrombosis, major amputation, major adverse limb events (MALE), and early mortality, although absolute numbers were small. Length of stay tended to be longer in the sarcopenic groups, and postoperative complications were consistently more frequent when data were available. Several studies did not report early cardiovascular events (stroke or myocardial infarction), reflecting variability in outcome definitions and follow-up protocols. Overall, the available evidence suggests a pattern of worse short-term postoperative outcomes in sarcopenic patients; however, the limited number of studies, small event counts, and substantial heterogeneity in outcome reporting preclude firm conclusions (Table 4).

**Table 4:** Postoperative complications and adverse events at 1 month.

Author	Graft thrombosis requiring reintervention		Postoperative complications		Length of stay (days)		Major amputation		MALE		Stroke		Myocardial infarction		MACCE		All-cause mortality	
	S	NS	S	NS	S	NS	S	NS	S	NS	S	NS	S	NS	S	NS	S	NS
Chikata et al. (22)	NA	NA	NA	NA	NA	NA	*NA*	*NA*	*NA*	*NA*	*NA*	*NA*	*NA*	*NA*	*NA*	*NA*	*NA*	*NA*
Söderlund et al. (20)	NA	NA	NA	NA	NA	NA	*NA*	*NA*	*NA*	*NA*	*NA*	*NA*	*NA*	*NA*	*NA*	*NA*	*NA*	*NA*
Selçuk et al. (24)	4/82	2/135	NA	NA	7[5–13]	6[4–11.5]	*6/82*	*6/135*	*10/82*	*8/135*	*1/82*	*0*	*3/82*	*0*	*NA*	*NA*	*7/82*	*1/135*
Pereira-Neves et al. (19)	NA	NA	17	21	7 [3–23]	8 [6.25–11.50]	*NA*	*NA*	*3/26*	*4/24*	*NA*	*NA*	*NA*	*NA*	*4/15*	*4/41*	*1/15*	*3/41*
Taniguchi et al. (23)	NA	NA	NA	NA	NA	NA	*NA*	*NA*	*NA*	*NA*	*NA*	*NA*	*NA*	*NA*	*NA*	*NA*	*NA*	*NA*
Juszczak et al. (21)	NA	NA	NA	NA	9 [4.0–28.0]	6 [4.0–9.0]	*NA*	*NA*	*NA*	*NA*	*NA*	*NA*	*NA*	*NA*	*NA*	*NA*	*2/63*	*4/190*
Nyers et al. (18)	NA	NA	NA	NA	NA	NA	*NA*	*NA*	*NA*	*NA*	*NA*	*NA*	*NA*	*NA*	*NA*	*NA*	*NA*	*NA*

MACCE, major adverse cardiovascular and cerebrovascular events; MALE, major adverse limb events; NA, unavailable data; NS, non-sarcopenic; S, sarcopenic.

Long-term outcomes were reported inconsistently, with follow-up ranging from 12 months to nearly 6 years. Across the studies providing stratified data, sarcopenic patients consistently demonstrated higher all-cause mortality compared with non-sarcopenic counterparts, and in some cohorts showed increased rates of major adverse cerebro- and cardiovascular events (MACCE), MALE, and major amputation. Although definitions and follow-up durations differed, the available evidence suggests an association between sarcopenia and worse long-term outcomes, particularly all-cause mortality. However, the limited number of studies and substantial heterogeneity in the reporting of limb-related outcomes preclude firm conclusions regarding their association with reduced psoas muscle area (Table 5).

**Table 5:** Postoperative complications and adverse events at 1 year.

Author	Follow-up	All-cause mortality		MACCE		MALE		Major amputation	
		S	NS	S	NS	S	NS	S	NS
Chikata et al. (22)	NA	26/162	7/162	NA	NA	NA	NA	NA	NA
Söderlund et al. (20)	71 months	13/225	9/224	NA	NA	NA	NA	NA	NA
Selçuk et al. (24)	1 month	NA	NA	NA	NA	NA	NA	NA	NA
Pereira-Neves et al. (19)	20 months	7/15	3/41	8/15	4/41	6/26	4/24	NA	NA
Taniguchi et al. (23)	14 months	24/50	8/25	NA	NA	NA	NA	27/50	8/25
Juszczak et al. (21)	21 months	18/63	22/190	NA	NA	NA	NA	NA	NA
Nyers et al. (18)	12 months	NA	NA	NA	NA	NA	NA	53/94	45/94

MACCE, major adverse cardiovascular and cerebrovascular events; MALE, major adverse limb events; NA, unavailable data; NS, non-sarcopenic; S, sarcopenic.

Covariate reporting across studies was heterogeneous, with only a subset providing adjusted models (Supplementary Table S6).

### Main findings and meta-analysis

**Figure 2:**
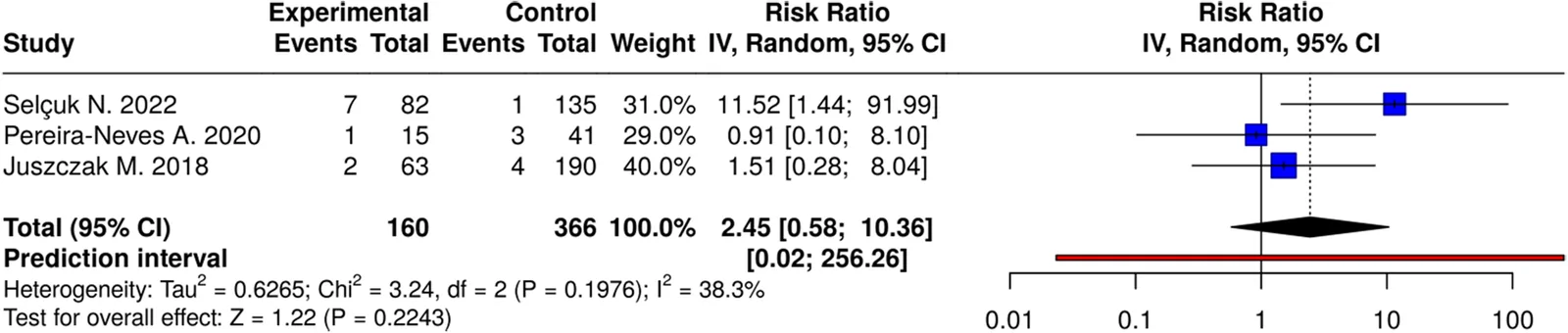
Forest plot of 1-month mortality comparing low vs. high psoas major muscle area. The experimental group corresponds to patients with low psoas muscle area (sarcopenic), whereas the control group corresponds to patients with high psoas muscle area (non-sarcopenic). CI, confidence interval; IV, inverse variance; MH, Mantel-Haenszel.

Three studies (19, 21, 24) reporting 1-month mortality were included in the quantitative synthesis. Individually, the effect estimates demonstrated substantial variability, with risk ratios ranging from 0.91 to 11.52, reflecting heterogeneity in sample sizes and event rates. The pooled random-effects model did not show a statistically significant association between sarcopenia (reduced psoas muscle area) and 1-month mortality (RR = 2.45; 95% CI 0.58–10.36; *p* = 0.22), despite a point estimate suggesting an increased risk. Between-study heterogeneity was moderate (*I*^2^ = 38.3%), and the prediction interval was wide (0.02–256.26), indicating substantial uncertainty around the expected effect in future settings. Overall, current evidence does not support a statistically significant association between low psoas muscle area and short-term mortality, but the wide confidence intervals suggest the analysis may be underpowered (Figure 2 and Supplementary Figure S1).

**Figure 3:**
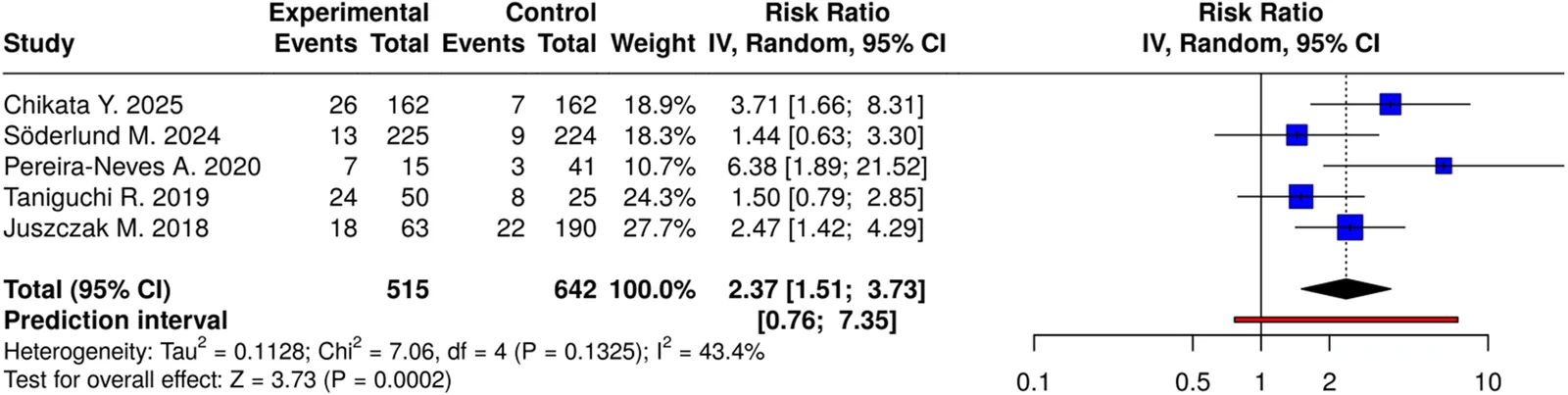
Forest plot of 1-year mortality comparing low vs. high psoas major muscle area. The experimental group corresponds to patients with low psoas muscle area (sarcopenic), whereas the control group corresponds to patients with high psoas muscle area (non-sarcopenic). CI, confidence interval; IV, inverse variance; MH, Mantel-Haenszel.

Five studies (19–23) contributed data to the 1-year mortality analysis. Across individual cohorts, sarcopenic patients consistently demonstrated higher mortality risk, with study-level risk ratios ranging from 1.44 to 6.38. The pooled random-effects model showed a significant association between reduced psoas muscle area and increased 1-year mortality (RR = 2.37; 95% CI 1.51–3.73; *p* < 0.001). Between-study heterogeneity was moderate (*I*^2^ = 43.4%), and although the prediction interval was wide (0.76–7.35), the overall direction of effect remained stable across studies. Overall, these results support a potential role for sarcopenia as a prognostic marker of 1-year mortality in patients undergoing lower-limb revascularization (Figure 3, Supplementary Figure S2). According to the GRADE approach, the certainty of evidence for the primary outcome (1-year all-cause mortality) was rated as low. A summary of the GRADE assessment is provided in Supplementary Table S2. A sensitivity analysis excluding studies without multivariable adjustment (Pereira-Neves et al. and Taniguchi et al.) yielded results comparable to the primary analysis (RR = 2.40; 95% CI 1.50–3.83; *p* < 0.001), with lower between-study heterogeneity (*I*^2^ = 23.2%). These findings suggest that the observed association was not materially influenced by the inclusion of studies without multivariable adjustment (Supplementary Figure S3). To explore the potential influence of extreme-category comparisons, an additional sensitivity analysis was performed in studies reporting tertile- or quartile-based classifications by comparing the lowest psoas category with all remaining categories. The results remained consistent with the primary analysis (RR = 2.29; 95% CI 1.62–3.24; *p* < 0.001), with lower between-study heterogeneity (*I*^2^ = 26.3%). These findings suggest that the observed association was not driven solely by comparisons between extreme categories and further support the robustness of the primary results (Supplementary Figure S4).

Only two studies reported limb-related events at 1 month (19, 24), whereas only one reported limb-related events at 1 year (19), precluding a pooled analysis across comparable follow-up time points. Specifically, Selçuk et al. reported 1-month data on graft thrombosis requiring reintervention, major amputation, and MALE, while Pereira-Neves et al. was the only study to report MALE at 1 year (20-month follow-up). Given the limited number of studies, differences in outcome definitions, and variability in follow-up duration, the available evidence regarding limb-related outcomes remains insufficient, and no firm conclusions can be drawn regarding the association between reduced psoas muscle area and limb-related events.

### Study quality

**Figure 4:**
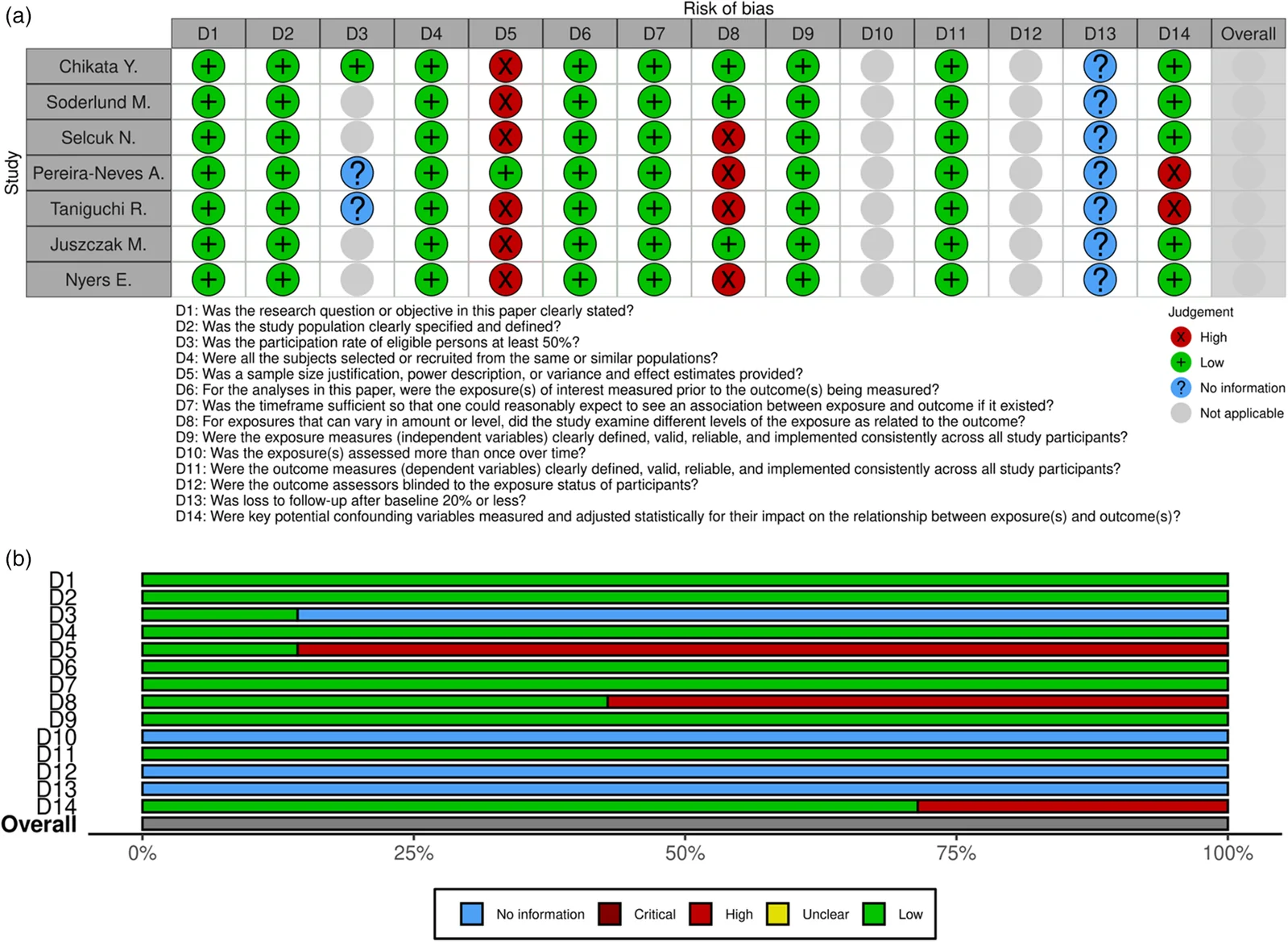
Risk of bias assessment for the cohort studies **(a)** summary of risk-of-bias judgments for each individual cohort study **(b)** domain-level judgments for cohort studies.

The risk of bias for the included studies is shown in Figure 4. The risk of bias for each observational cohort is individually displayed in Figure 4a. The overall judgement per evaluated item regarding observational cohorts is shown in Figure 4b.

All included studies were observational. Overall, most domains across the studies show a low risk of bias, but several specific domains consistently raised concerns. The items most frequently associated with higher risk were sample size justification, power description, variance and effect estimates (D5), assessment of exposures that can vary in amount or level (D8), and measurement and statistical adjustment for key potential confounding variables (D14). In addition, several studies provided little or no information for domains such as participation rate/response (D3) and loss to follow-up (D13), which limits confidence in those areas. In sum, while the majority of assessed domains appear adequate, shortcomings in sample-size reporting, exposure assessment, and confounder control remain important weaknesses across these observational studies.

### Publication bias

Funnel plots for 1-month and 1-year mortality are presented in Supplementary Figures S1, S2. Visual inspection did not suggest marked asymmetry, although assessment of publication bias was limited by the small number of included studies. Formal asymmetry testing was therefore interpreted cautiously.

## Discussion

In this systematic review and meta-analysis, lower psoas muscle area, as an imaging-derived surrogate of sarcopenia, was associated with adverse long-term outcomes in patients undergoing lower-limb revascularization for peripheral arterial disease. Across seven observational cohorts comprising 2,290 patients, sarcopenic individuals had higher pooled 1-year mortality than non-sarcopenic counterparts. Although 1-month mortality did not reach statistical significance, possibly reflecting low event rates and limited power, the direction of effect was similar. Despite heterogeneity in anatomical level, imaging modality, and diagnostic thresholds used to define sarcopenia, the overall findings suggest that psoas morphometry may represent a prognostic marker for identifying higher-risk patients. However, the wide prediction interval crossing the null value indicates that the magnitude and consistency of this association may vary across different clinical settings, and the findings should therefore be interpreted with appropriate caution. The selection of 1-month and 1-year outcomes was based on their clinical relevance and consistent reporting across the included studies. One-month mortality primarily reflects perioperative risk and early postoperative resilience, whereas 1-year mortality provides a broader assessment of the longer-term prognostic implications of sarcopenia beyond the immediate surgical period.

Regarding 1-month mortality, several studies have evaluated CT-derived psoas muscle measurements as a surrogate of sarcopenia and their association with short-term mortality, with inconsistent results. Brzeszczyński et al., in a systematic review of seven studies with a total of 2,080 patients, reported that CT-derived markers of reduced psoas muscle mass were associated with increased 1-month postoperative mortality following emergency laparotomy (25). Similarly, studies in cardiovascular surgery, including a prospective cohort of 1,076 patients by Kofler et al., have reported associations with early mortality after transcatheter aortic valve implantation (TAVI) (26). In contrast, other large retrospective cohorts and registry-based analyses have failed to demonstrate an independent association between low psoas muscle area and short-term mortality (27–29). These findings align with the present meta-analysis, which found that reduced psoas muscle area was associated with a higher point estimate of 1-month mortality but did not reach statistical significance. The observed heterogeneity and wide confidence and prediction intervals suggest substantial imprecision, likely reflecting differences in patient populations, sarcopenia definitions, imaging methodologies, and limited event rates.

With respect to 1-year mortality, the existing literature suggests a more consistent association between CT-derived psoas muscle measurements and adverse outcomes, supporting a potential prognostic role of sarcopenia beyond the early postoperative period. Several cohorts have reported increased 1-year mortality among patients with low psoas muscle area, supporting the potential prognostic relevance of sarcopenia at this time horizon (14, 30). Xu et al. reported a hazard ratio of 0.689 in a retrospective cohort of 116 patients, indicating that low PMI was associated with higher 1-year mortality among young male patients with acute-on-chronic liver failure (ACLF), independent of Model for End-Stage Liver Disease (MELD) score (14). Caio et al., in a prospective cohort of 617 patients undergoing elective or urgent arterial vascular surgery, reported a hazard ratio of 1.76, also showing that low PMA was associated with 1-year mortality (30).

Alternative imaging-based approaches to body composition assessment have also been explored in patients with peripheral arterial disease, including measures of muscle quality such as psoas muscle density (PMD) (31). In the present review, Pereira-Neves et al. evaluated both total psoas area and PMD as prognostic markers (19). While reduced total psoas area was associated with higher rates of MACCE and all-cause mortality, PMD did not demonstrate significant associations with these outcomes (19). These findings highlight the potential differences between muscle quantity and muscle quality measures and underscore the need for further studies to clarify their relative prognostic value in patients with PAD.

Only two studies reported early (1-month) limb events, capturing perioperative complications such as graft thrombosis, major amputation and MALE (19, 24), whereas only one study assessed later MALE at approximately 1 year (19), reflecting a distinct pathophysiological window influenced by disease progression, durability of revascularization, and long-term patient factors. Selçuk et al. reported a higher incidence of major adverse cardiovascular events (MACE) but not of MALE (24). Pereira-Neves et al. showed that neither TPA nor PMD revealed statistical significance for MALE (19). These outcomes are not interchangeable, as early events are more closely related to technical success and perioperative resilience, while late events integrate frailty, comorbidity burden, and sustained ischemic risk (32). Consequently, the inability to align limb outcomes at comparable time points likely attenuates effect estimates and limits quantitative synthesis.

This study has several limitations worth noting. First, only a small number of studies were eligible for inclusion, and most had limited sample sizes without formal sample size justification or power calculations, which may have reduced the precision of the pooled estimates. Second, heterogeneity existed across studies in baseline patient characteristics, sarcopenia definitions, and methodological approaches. Meta-regression was not performed because of the limited number of included studies. Subgroup analyses according to imaging modality (CT vs. MRI) or sarcopenia definition were not performed because of the limited number of studies available within each subgroup, which would have resulted in unreliable estimates. In addition, few secondary short- and long-term outcomes were consistently reported, limiting the ability to assess associations with other cardiovascular and limb-related events. The inconsistent reporting of limb-related outcomes also precluded pooled analyses for these endpoints. Information regarding physical function and functional status was also inconsistently reported across the included studies, limiting further evaluation of the relationship between psoas morphometry, functional capacity, and clinical outcomes. Similarly, frailty indices were rarely reported, preventing assessment of their potential influence on the observed associations. Finally, all included studies were retrospective observational cohorts, which introduces inherent risks of residual confounding and selection bias.

### Future directions

In light of these considerations and the findings of the present systematic review and meta-analysis, a more comprehensive, patient-centered approach to the management of peripheral arterial disease may be warranted. Beyond revascularization alone, selected patients may benefit from structured physical and rehabilitation medicine (PRM) programs, including supervised exercise therapy and resistance training, as well as targeted nutritional interventions to optimize protein and energy intake, prevent or mitigate sarcopenia, and improve overall functional reserve. While emerging evidence suggests that such multimodal strategies may have a favorable impact on functional capacity and clinical outcomes (10, 33), the current body of literature remains limited and heterogeneous. Therefore, further well-designed prospective studies and randomized controlled trials are required to clarify the effectiveness, optimal timing, and patient selection criteria for rehabilitation and nutritional interventions in this population.

This systematic review highlights the need for large prospective cohorts and registries specifically designed to evaluate sarcopenia in patients undergoing lower-limb revascularization, validate these findings, and address remaining knowledge gaps.

## Conclusion

This systematic review and meta-analysis suggests that lower psoas muscle area, as an imaging surrogate of sarcopenia, is associated with increased 1-year mortality in patients with peripheral arterial disease undergoing lower-limb revascularization. Although short-term mortality findings were inconclusive, psoas morphometry may represent a candidate prognostic marker that requires prospective validation before routine clinical implementation. These findings raise the possibility that sarcopenia assessment may help identify vulnerable patients who could benefit from optimized perioperative care and targeted nutritional or rehabilitative strategies. Further prospective studies and standardized definitions are needed before routine integration into vascular surgical practice can be recommended.

## Data Availability

The original contributions presented in the study are included in the article/Supplementary Material, further inquiries can be directed to the corresponding author.
