# Supplementary material for: Psoas Major muscle area as a prognostic marker in peripheral arterial disease: a systematic review and meta-analysis
Source: Front Surg. 2026 Jul 15;13:1879694. doi: 10.3389/fsurg.2026.1879694 (PMC13415353; doi:10.3389/fsurg.2026.1879694)
Supplement: Supplementary file 1 [file Table1.docx]

Table 1S. Search query

| **Bibliographic source** | **Search term** | **No of reports**  4/8/2025 |
| --- | --- | --- |
| MEDLINE | (sarcopenia OR “psoas muscle area” OR “psoas muscle loss” OR “lean psoas muscle” OR “psoas muscle attenuation” OR “muscle mass” OR “muscle area” OR “muscular atrophy” OR “Psoas Major Muscle area”)  AND  (“arterial occlusive diseases" OR “peripheral vascular disease” OR “peripheral artery disease” OR “peripheral arterial occlusive disease” OR “lower extremity arterial disease" OR "lower limb ischemia" OR "chronic limb-threatening ischemia" OR "critical limb ischemia") | 126 |
| Web of Science | (sarcopenia OR “psoas muscle area” OR “psoas muscle loss” OR “lean psoas muscle” OR “psoas muscle attenuation” OR “muscle mass” OR “muscle area” OR “muscular atrophy” OR “Psoas Major Muscle area”)  AND  (“arterial occlusive diseases" OR “peripheral vascular disease” OR “peripheral artery disease” OR “peripheral arterial occlusive disease” OR “lower extremity arterial disease" OR "lower limb ischemia" OR "chronic limb-threatening ischemia" OR "critical limb ischemia") | 154 |
| SCOPUS | (sarcopenia OR “psoas muscle area” OR “psoas muscle loss” OR “lean psoas muscle” OR “psoas muscle attenuation” OR “muscle mass” OR “muscle area” OR “muscular atrophy” OR “Psoas Major Muscle area”)  AND  (“arterial occlusive diseases" OR “peripheral vascular disease” OR “peripheral artery disease” OR “peripheral arterial occlusive disease” OR “lower extremity arterial disease" OR "lower limb ischemia" OR "chronic limb-threatening ischemia" OR "critical limb ischemia") | 244 |

Table 2S. Characteristics of included studies: design, setting and population

| **Author** | **Journal** | **Publication Year** | **Study Design** | **Study Center** | **Continent** | **Recruitment Time** | **Sample Size (patients)** | **No. Interventions** | **GRADE** |
| --- | --- | --- | --- | --- | --- | --- | --- | --- | --- |
| *Chikata Y. et al.* | Catheterization and Cardiovascular Interventions | 2025 | Retrospective cohort | Juntendo University Hospital, Japan | Asia | Between January 2009 and March 2020 | 591 | 591 | 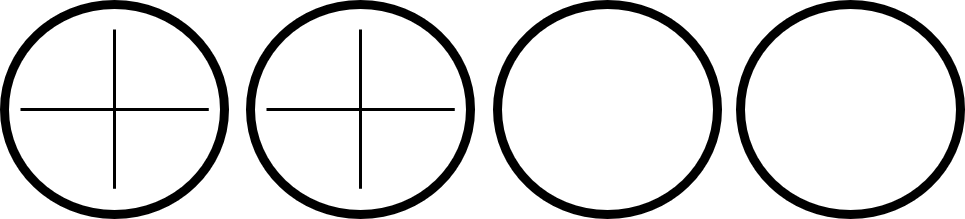  Low |
| *Söderlund M. et al.* | Scandinavian Journal of Surgery | 2024 | Retrospective cohort | Tampere University Hospital, Finland | Europe | Between 1 January 2010 and 27 November 2020 | 899 | 899 | 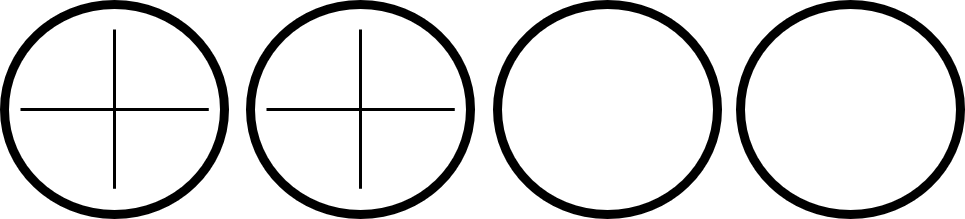  Low |
| *Selçuk N. et al.* | Vascular | 2022 | Retrospective cohort | Tertiary referral center for cardiovascular diseases, Turkey | Asia | Between October 2015 and December 2020 | 217 | 217 | 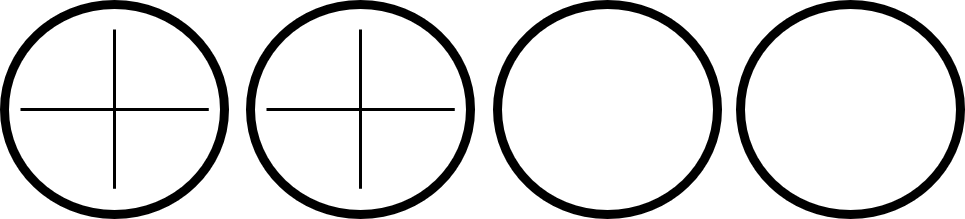  Low |
| *Pereira-Neves A. et al.* | Turkish Journal of Thoracic and Cardiovascular Surgery | 2020 | Retrospective cohort | Centro Hospitalar Universitário de São João and Centro Hospitalar do Tâmega e Sousa, Portugal | Europe | Between January 2013 and July 2019 | 57 | 57 | 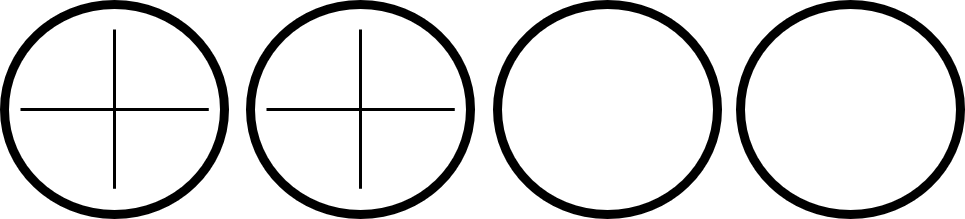  Low |
| *Taniguchi R. et al.* | Annals of Vascular Diseases | 2019 | Retrospective cohort | Saitama Medical Center, Saitama Medical University, Japan | Asia | Between 2011 and 2015 | 75 | 75 | 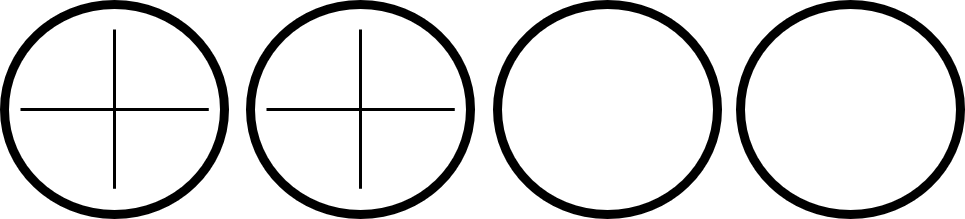  Low |
| *Juszczak M. et al.* | Journal of Vascular Surgery | 2018 | Retrospective cohort | Oxford University Hospitals NHS Foundation Trust, Liverpool Vascular and Endovascular Services (LiVES), and The Royal Oldham Hospital, United Kingdom | Europe | Between January 2013 and December 2014 | 263 | 263 | 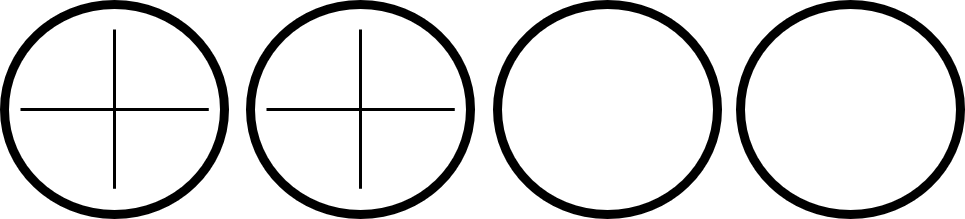  Low |
| *Nyers E. et al.* | Journal of Vascular Surgery | 2017 | Retrospective cohort | Medical University of South Carolina and Ralph H. Johnson VA Medical Center, United States | America | Between June 16, 2010, and May 19, 2016 | 188 | 188 | 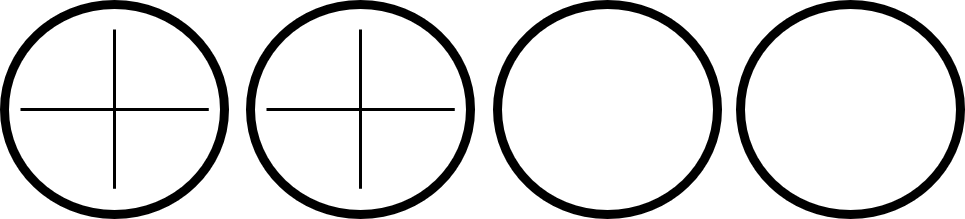  Low |

Table 3S. Statin and antiplatelet therapy use

| **Author** | **Statins n** | **Antiplatelet therapy n** |
| --- | --- | --- |
| *Chikata Y. et al.* | 298 | NA |
| *Söderlund M. et al.* | 579 | NA |
| *Selçuk N. et al.* | NA | NA |
| *Pereira-Neves A. et al.* | NA | NA |
| *Taniguchi R. et al.* | NA | NA |
| *Juszczak M. et al.* | 226 | 219 |
| *Nyers E. et al.* | NA | NA |

NA - unavailable data

Table 4S. Psoas muscle area and index measurements

| **Author** | **Psoas area (mean or median)** | **Psoas muscle index (PMI) - men** | **Psoas muscle index (PMI) - women** |
| --- | --- | --- | --- |
| *Chikata Y. et al.* | NA | NA | NA |
| *Söderlund M. et al.* | All patients - 6.9 cm2. The psoas muscle surface areas were larger in men (mean = 7.6 cm2) compared to women (mean = 5.3 cm2) | NA | NA |
| *Selçuk N. et al.* | NA | 7.45 cm2/m2 | 4.49 cm2/m2 |
| *Pereira-Neves A. et al.* | Mean was 2,447±491.4 (range, 1,285 to 3,459) mm2 | NA | NA |
| *Taniguchi R. et al.* | NA | 8.66 cm2/m2 | 6.38 cm2/m2 |
| *Juszczak M. et al.* | Median was 1,964.0 mm2 and it was significantly greater in men (2,191.5 mm2; IQR, 1,793.7-2,619.7 mm2) than in women (1,476.0 mm2; IQR,1,287.0-1,753.0 mm2). | NA | NA |
| *Nyers E. et al.* | Women - 18.1 cm2 and men - 26.8 cm2 | NA | NA |

IQR - interquartile range; NA - unavailable data

Table 5S. Definitions of MACCE and MALE

| **Author** | **MACCE definition** | **MALE definition** |
| --- | --- | --- |
| *Chikata Y. et al.* | NA | NA |
| *Söderlund M. et al.* | NA | NA |
| *Selçuk N. et al.* | NA | Major adverse limb events included above-the-ankle amputations after revascularization or graft thrombosis requiring reintervention. |
| *Pereira-Neves A. et al.* | Composite outcome of stroke, myocardial infarction, coronary reintervention, acute heart failure, and all-cause mortality | Defined as loss of primary patency (interventions for assisted primary patency, secondary patency or loss of patency without reintervention), and major amputation |
| *Taniguchi R. et al.* | NA | NA |
| *Juszczak M. et al.* | NA | NA |
| *Nyers E. et al.* | NA | NA |

MACCE - major adverse cardiovascular and cerebrovascular events; MALE - major adverse limb events; NA - unavailable data

| **Author** | **Covariables used in adjusted models** |
| --- | --- |
| *Chikata Y. et al.* | TCBI |
| *Söderlund M. et al.* | Men - Age; Stroke/TIA; Pulmonary disease; Renal insufficiency; Fontaine classification IIb; Fontaine classification IV; Urgent procedure  Women - Age; Stroke/TIA; Renal insufficiency; Fontaine classification IV; |
| *Selcuk N. et al.* | NA |
| *Pereira-Neves A. et al.* | NA |
| *Taniguchi R. et al.* | NA |
| *Juszczak M. et al.* | Emergency Status; Fontaine Stage; Asa Score |
| *Nyers E. et al.* | Age; Non-African American race; Open bypass |

Table 6S. Statistically significant covariates in multivariable models

ASA - American Society of Anesthesiologists; NA - unavailable data; TCBI - triglycerides, total cholesterol, and body weight index; TIA - transient ischemic attack
