# Supplementary material for: Psoas Major muscle area as a prognostic marker in peripheral arterial disease: a systematic review and meta-analysis
Source: Front Surg. 2026 Jul 15;13:1879694. doi: 10.3389/fsurg.2026.1879694 (PMC13415353; doi:10.3389/fsurg.2026.1879694)

Study	Experimental Events	Experimental Total	Control Events	Control Total	Weight	Risk Ratio IV, Random, 95% CI
Chikata Y. 2025	26	162	7	162	27.1%	3.71 [1.66; 8.31]
Söderlund M. 2024	13	225	9	224	25.8%	1.44 [0.63; 3.30]
Juszczak M. 2018	18	63	22	190	47.1%	2.47 [1.42; 4.29]
**Total (95% CI)**		**450**		**576**	**100.0%**	**2.40 [1.50; 3.83]**
**Prediction interval**						**[0.62; 9.20]**

Heterogeneity: $Tau^2 = 0.0409$; $Chi^2 = 2.60$, df = 2 (P = 0.2722); $I^2 = 23.2\%$
Test for overall effect: Z = 3.67 (P = 0.0002)

Risk Ratio
IV, Random, 95% CI

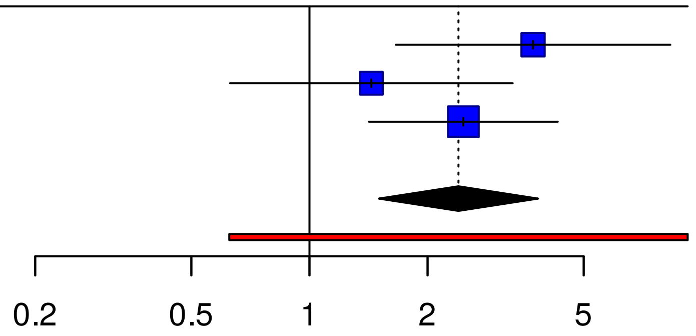

Supplement: Supplementary file 2 [file Datasheet1.pdf]
