# Supplementary figures and images for: Psoas Major muscle area as a prognostic marker in peripheral arterial disease: a systematic review and meta-analysis
Source: Front Surg. 2026 Jul 15;13:1879694. doi: 10.3389/fsurg.2026.1879694 (PMC13415353; doi:10.3389/fsurg.2026.1879694)

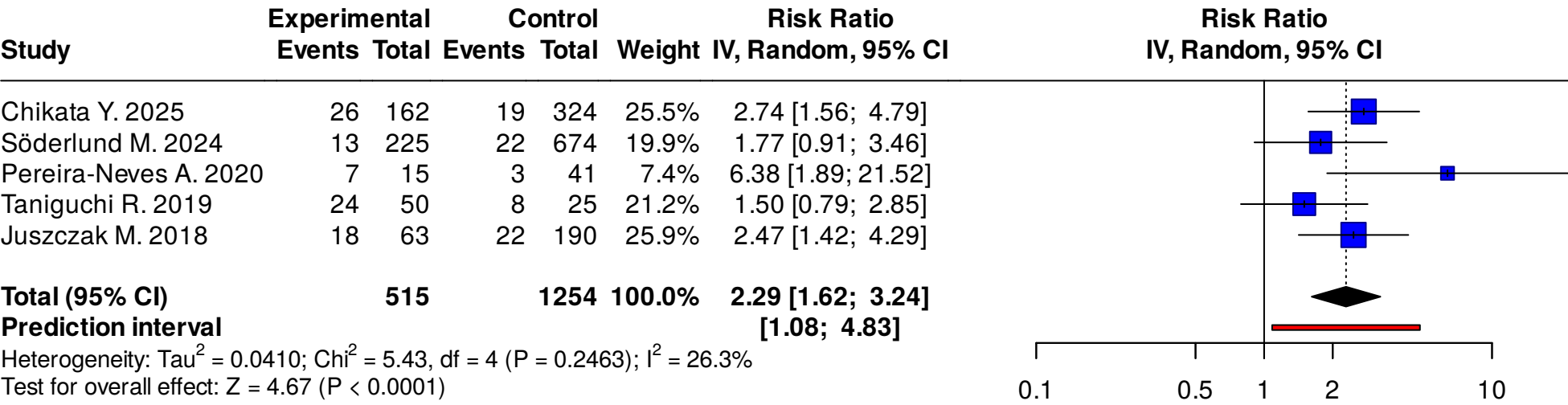

Supplement: Supplementary file 3 [file Datasheet2.pdf]

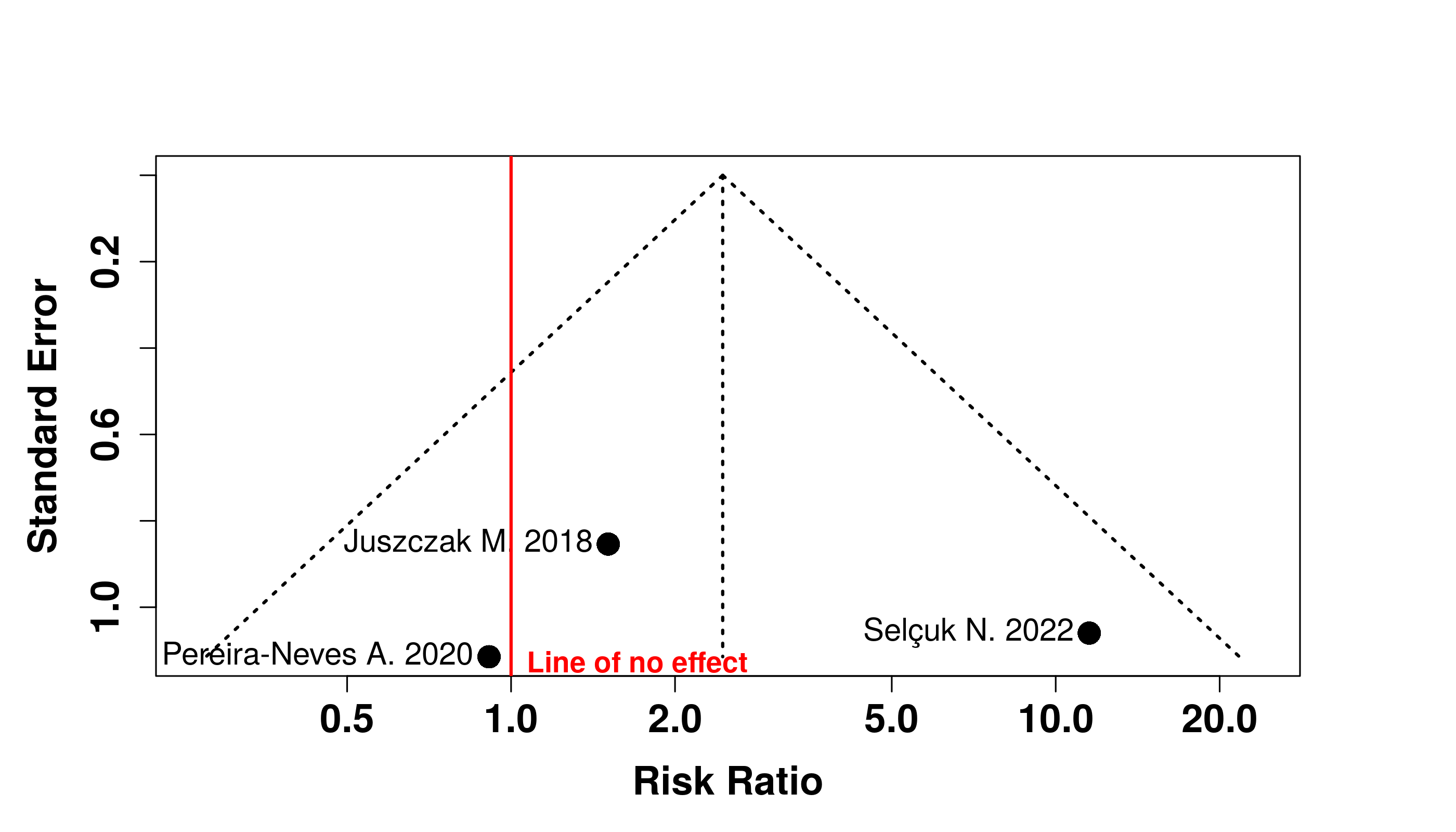

Supplement: Supplementary file 4 [file Image1.png]

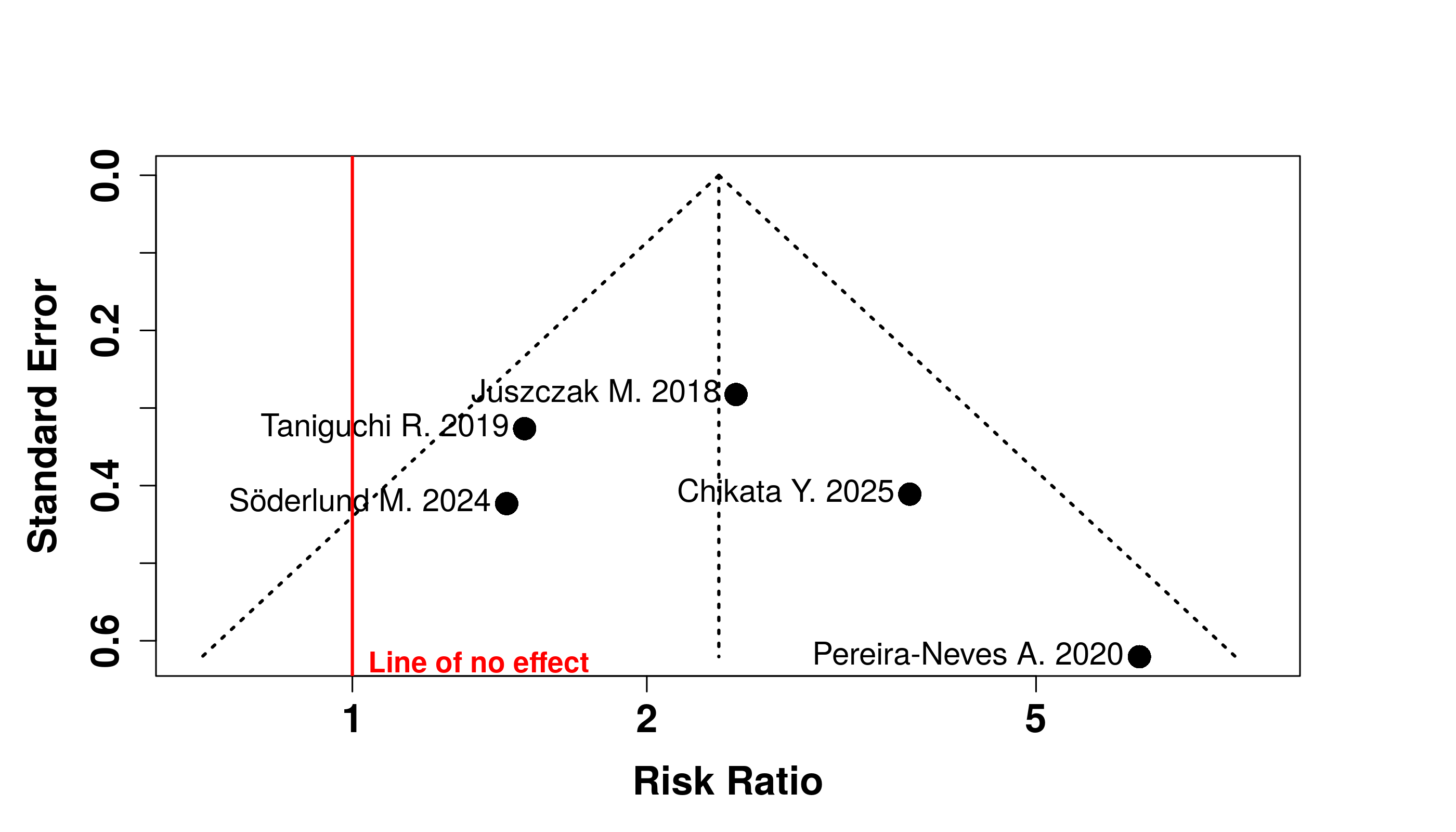

Supplement: Supplementary file 5 [file Image2.png]
